# 1-(Biphenyl-4-ylmethyl­idene)thio­semicarbazide monohydrate

**DOI:** 10.1107/S1600536810011888

**Published:** 2010-04-10

**Authors:** Rafael Mendoza-Meroño, Laura Menéndez-Taboada, Eva Fernández-Zapico, Santiago García-Granda

**Affiliations:** a Departamento de Química Física y Analítica, Facultad de Química, Universidad de Oviedo, C/ Julián Clavería, 8, 33006 Oviedo (Asturias), Spain

## Abstract

In the title compound, C_14_H_13_N_3_S·H_2_O, the thio­semicarbazide group is nearly planar, with a maximum deviation of 0.072 (2) Å from the ideal least-squares plane, and shows an *E* conformation. In the crystal packing, the water mol­ecules are involved in an extensive inter­molecular N—H⋯O hydrogen-bond network, assisted by O—H⋯S inter­actions, which link the independent mol­ecules into chains extended along *b* axis. An intra­molecular hydrogen N—H⋯N bond helps to stabilize the mol­ecular conformation.

## Related literature

For the biological activity and potential medical applications of thio­semicarbazides, see: West *et al.* (1991 ▶). For thio­semicarbazides as ligands, see: Kowol *et al.* (2007 ▶).
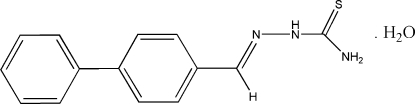


## Experimental

### 

#### Crystal data

C_14_H_13_N_3_S·H_2_O
*M* *_r_* = 273.36
Monoclinic, 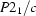

*a* = 14.428 (5) Å
*b* = 6.350 (5) Å
*c* = 15.276 (4) Å
β = 99.750 (5)°
*V* = 1379.3 (12) Å^3^
*Z* = 4
Cu *K*α radiation
μ = 2.05 mm^−1^
*T* = 293 K
0.28 × 0.22 × 0.19 mm

#### Data collection

Oxford Diffraction Xcalibur Gemini S diffractometer
Absorption correction: multi-scan (*CrysAlis PRO*; Oxford Diffraction, 2009 ▶) *T*_min_ = 0.659, *T*_max_ = 1.000
8823 measured reflections
2652 independent reflections
2208 reflections with *I* > 2σ(*I*)
*R*_int_ = 0.035

#### Refinement

*R*[*F* ^2^ > 2σ(*F* ^2^)] = 0.044
*wR*(*F* ^2^) = 0.145
*S* = 1.12
2652 reflections
233 parameters
H atoms treated by a mixture of independent and constrained refinement
Δρ_max_ = 0.34 e Å^−3^
Δρ_min_ = −0.32 e Å^−3^

### 

Data collection: *CrysAlis CCD* (Oxford Diffraction, 2009 ▶); cell refinement: *CrysAlis RED* (Oxford Diffraction, 2009 ▶); data reduction: *CrysAlis RED*; program(s) used to solve structure: *SIR92* (Altomare *et al.*, 1994 ▶); program(s) used to refine structure: *SHELXL97* (Sheldrick, 2008 ▶); molecular graphics: *ORTEP-3 for Windows* (Farrugia, 1997 ▶); software used to prepare material for publication: *WinGX* (Farrugia, 1999 ▶) and *PLATON* (Spek, 2009 ▶).

## Supplementary Material

Crystal structure: contains datablocks global, I. DOI: 10.1107/S1600536810011888/vm2023sup1.cif

Structure factors: contains datablocks I. DOI: 10.1107/S1600536810011888/vm2023Isup2.hkl

Additional supplementary materials:  crystallographic information; 3D view; checkCIF report

## Figures and Tables

**Table 1:** Hydrogen-bond geometry (Å, °)

*D*—H⋯*A*	*D*—H	H⋯*A*	*D*⋯*A*	*D*—H⋯*A*
N2—H14⋯O1	0.92 (3)	1.91 (3)	2.819 (3)	172 (3)
N3—H15⋯N1	0.91 (3)	2.14 (3)	2.585 (3)	110 (2)
N3—H16⋯S1^i^	0.86 (4)	2.59 (4)	3.422 (3)	163 (3)
O1—H17⋯S1^ii^	0.86 (5)	2.44 (5)	3.287 (3)	169 (4)
O1—H18⋯S1^iii^	0.77 (5)	2.60 (5)	3.352 (3)	164 (5)

Symmetry codes: (i) 
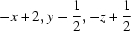
; (ii) 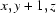; (iii) 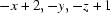.

**Fig. 1.**
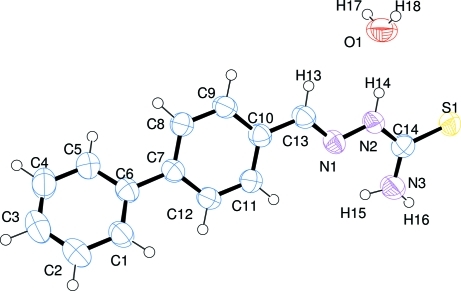
A view of (I), showing the atom-numbering scheme. Displacement ellipsoids are drawn at the 50% probability level.

**Fig. 2.**
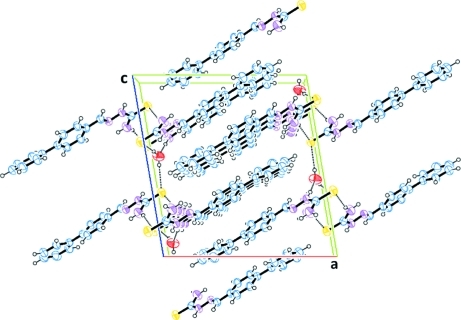
Packing diagram viewed parallel to the b axis. Hydrogen bonds and other intermolecular interactions are indicated by dashed lines.

## supplementary crystallographic information

### Comment

Thiosemicarbazides, a class of compounds possessing a wide spectrum of potential medicinal applications, have been studied for their antitumoral, antiviral, antibacterial, antimalarial, antifungal, anti-inflammatory and anti-HIV activities (West *et al.*, 1991). These properties are thought to arise from the metal-chelating ability of these ligands. In almost all cases, the ligands are bidentate and bind to the metal through the S and hydrazinic N atoms, although there are examples of them acting as monodentate ligands binding only through sulfur (Kowol *et al.*,  2007).

The title compound C_14_H_13_N_3_S.H_2_O was synthesized and its crystal structure is reported here. This compound is likely to have biomedical properties similar to other nitrogen-sulfur donor ligands. The asymmetric unit consists of a single molecule (I), shown in Figure 1. The thiosemicarbazide adopts an E conformation with a *trans* configuration observed about the C=N bond. The thiosemicarbazide moiety is planar, the C(14)—N(2) (1.341 (3) Å) and N(1)—N(2) (1.371 (3) Å) bond lengths imply significant electron delocalization and the C13/N1/N2/C14/S1 fragment is close to planar (max. deviation = 0.072 (2) Å). The dihedral angle between benzene ring C7/C8/C9/C10/C11/C12 and the moiety C13/N1/N2/C14/S1 is 4.67 (1)°. This value suggests that they are nearly coplanar, and π-electrons are delocalized in the benzaldehyde thiosemicarbazide fragment.

The water molecules are involved in an extensive intermolecular N(2)—H(14)···O(1) hydrogen bonds and O(1)—H(17)···S(1) interactions (Table 1), which link the molecules into chains extended along the *b* axis. Sulfur atom S(1) is also involved in N(3)—H(16)···S(1) intermolecular interactions, favoring the crystal grown in the *ac* plane. An intramolecular N(3)—H(15)···N(1) hydrogen bond (Table 1) contributes to stabilize the molecular conformation. The intermolecular distance value between ring centroids in the *b* axis direction (6.350 Å), suggests that there is no π-stacking interaction between parallel molecules (Figure 2).

### Experimental

A solution of 4-biphenylcarboxaldehyde (1.822 g, 0.01 mol) and thiosemicarbazide (0.91 g, 0.01 mol) in absolute methanol (50 ml) was refluxing for 4 h, in the presence of *p*-toluenesulfonic acid (0.005 g) as catalyst, with continuous stirring. Completeness of the reaction was TLC controlled indicating the disappearance of the aldehyde spot. On cooling to room temperature the precipitate was filtered off, washed with copious cold methanol and dried in air (yield: 1.581 g, 61%; m.p. 475 K). Yellow single crystals compound were obtained after recrystallization from a solution of chloroform/methanol (3:7 *v*/*v*) after 10 days at room temperature.

### Refinement

At the end of the refinement the highest peak in the electron density was 0.3350 e Å ^-3^, while the deepest hole was -0.3210 e Å ^-3^.

### Crystal data

**Table:**

C_14_H_13_N_3_S·H_2_O	*F*(000) = 576
*M**_r_* = 273.36	*D*_x_ = 1.316 Mg m^−^^3^
Monoclinic, *P*2_1_/*c*	Melting point: 475 K
Hall symbol: -P 2ybc	Cu *K*α radiation, λ = 1.54180 Å
*a* = 14.428 (5) Å	Cell parameters from 3594 reflections
*b* = 6.350 (5) Å	θ = 3.9–71.2°
*c* = 15.276 (4) Å	µ = 2.05 mm^−^^1^
β = 99.750 (5)°	*T* = 293 K
*V* = 1379.3 (12) Å^3^	Needle, yellow
*Z* = 4	0.28 × 0.22 × 0.19 mm

### Data collection

**Table:**

Oxford Diffraction Xcalibur Gemini S diffractometer	2652 independent reflections
Radiation source: Enhance (Cu) X-ray Source	2208 reflections with *I* > 2σ(*I*)
graphite	*R*_int_ = 0.035
Detector resolution: 10.2673 pixels mm^-1^	θ_max_ = 71.3°, θ_min_ = 5.9°
ω scans	*h* = −17→17
Absorption correction: multi-scan (*CrysAlis PRO*; Oxford Diffraction, 2009)	*k* = −6→7
*T*_min_ = 0.659, *T*_max_ = 1.000	*l* = −17→18
8823 measured reflections	

### Refinement

**Table:**

Refinement on *F*^2^	Secondary atom site location: difference Fourier map
Least-squares matrix: full	Hydrogen site location: inferred from neighbouring sites
*R*[*F*^2^ > 2σ(*F*^2^)] = 0.044	H atoms treated by a mixture of independent and constrained refinement
*wR*(*F*^2^) = 0.145	*w* = 1/[σ^2^(*F*_o_^2^) + (0.0724*P*)^2^ + 0.4128*P*] where *P* = (*F*_o_^2^ + 2*F*_c_^2^)/3
*S* = 1.12	(Δ/σ)_max_ = 0.001
2652 reflections	Δρ_max_ = 0.34 e Å^−^^3^
233 parameters	Δρ_min_ = −0.32 e Å^−^^3^
0 restraints	Extinction correction: *SHELXL97* (Sheldrick, 2008), Fc^*^=kFc[1+0.001xFc^2^λ^3^/sin(2θ)]^-1/4^
Primary atom site location: structure-invariant direct methods	Extinction coefficient: 0.0022 (5)

### Special details

**Table:**

Experimental. CrysAlisPro, Oxford Diffraction Ltd., Version 1.171.34.9 (release 08-12-2009 CrysAlis171 .NET)(compiled Dec 8 2009,17:31:18). Empirical absorption correction using spherical harmonics,implemented in SCALE3 ABSPACK scaling algorithm.
Geometry. All esds (except the esd in the dihedral angle between two l.s. planes) are estimated using the full covariance matrix. The cell esds are taken into account individually in the estimation of esds in distances, angles and torsion angles; correlations between esds in cell parameters are only used when they are defined by crystal symmetry. An approximate (isotropic) treatment of cell esds is used for estimating esds involving l.s. planes.
Refinement. Refinement of *F*^2^ against ALL reflections. The weighted *R*-factor wR and goodness of fit *S* are based on *F*^2^, conventional *R*-factors *R* are based on *F*, with *F* set to zero for negative *F*^2^. The threshold expression of *F*^2^ > σ(*F*^2^) is used only for calculating *R*-factors(gt) etc. and is not relevant to the choice of reflections for refinement. *R*-factors based on *F*^2^ are statistically about twice as large as those based on *F*, and *R*- factors based on ALL data will be even larger.

### Fractional atomic coordinates and isotropic or equivalent isotropic displacement parameters (Å^2^)

**Table:**

	*x*	*y*	*z*	*U*_iso_*/*U*_eq_	
S1	1.03245 (4)	−0.35800 (9)	0.35948 (4)	0.0569 (2)	
N2	0.87783 (13)	−0.1308 (3)	0.31541 (13)	0.0506 (5)	
N1	0.79162 (12)	−0.0958 (3)	0.26420 (12)	0.0512 (5)	
O1	0.95339 (19)	0.1981 (4)	0.42955 (18)	0.0826 (7)	
C9	0.62409 (16)	0.3346 (4)	0.22359 (17)	0.0554 (6)	
C14	0.92353 (15)	−0.3091 (4)	0.30268 (15)	0.0518 (5)	
N3	0.87879 (17)	−0.4428 (4)	0.24280 (16)	0.0687 (6)	
C7	0.48473 (14)	0.2361 (4)	0.12056 (15)	0.0490 (5)	
C13	0.75391 (15)	0.0839 (4)	0.27165 (16)	0.0526 (5)	
C10	0.66283 (15)	0.1342 (3)	0.21913 (15)	0.0506 (5)	
C11	0.61170 (16)	−0.0131 (4)	0.16261 (17)	0.0546 (5)	
C8	0.53638 (16)	0.3841 (4)	0.17552 (16)	0.0543 (6)	
C6	0.39026 (15)	0.2860 (4)	0.07042 (15)	0.0516 (5)	
C12	0.52453 (16)	0.0367 (4)	0.11508 (16)	0.0545 (6)	
C5	0.36951 (18)	0.4859 (4)	0.03497 (19)	0.0639 (6)	
C1	0.31911 (17)	0.1354 (4)	0.05726 (18)	0.0609 (6)	
C2	0.22957 (19)	0.1843 (5)	0.01258 (19)	0.0707 (7)	
C4	0.2796 (2)	0.5341 (5)	−0.0100 (2)	0.0748 (8)	
C3	0.2100 (2)	0.3841 (6)	−0.02047 (18)	0.0728 (8)	
H8	0.5121 (18)	0.525 (5)	0.1814 (18)	0.063 (7)*	
H12	0.4912 (18)	−0.064 (4)	0.0746 (17)	0.060 (7)*	
H5	0.421 (2)	0.590 (5)	0.041 (2)	0.078 (9)*	
H1	0.3329 (17)	−0.005 (4)	0.0831 (18)	0.060 (7)*	
H9	0.6583 (17)	0.436 (4)	0.2642 (17)	0.057 (7)*	
H13	0.7819 (17)	0.187 (4)	0.3128 (17)	0.052 (6)*	
H11	0.638 (2)	−0.147 (4)	0.1583 (19)	0.066 (8)*	
H3	0.146 (2)	0.420 (5)	−0.053 (2)	0.085 (9)*	
H4	0.267 (2)	0.679 (6)	−0.036 (2)	0.094 (10)*	
H2	0.180 (2)	0.076 (6)	0.004 (2)	0.094 (11)*	
H14	0.907 (2)	−0.033 (5)	0.3552 (19)	0.066 (8)*	
H15	0.825 (2)	−0.392 (5)	0.211 (2)	0.072 (8)*	
H16	0.907 (2)	−0.553 (6)	0.229 (2)	0.082 (9)*	
H17	0.975 (3)	0.304 (8)	0.404 (3)	0.129 (16)*	
H18	0.959 (3)	0.210 (7)	0.481 (3)	0.120 (17)*	

### Atomic displacement parameters (Å^2^)

**Table:**

	*U*^11^	*U*^22^	*U*^33^	*U*^12^	*U*^13^	*U*^23^
S1	0.0468 (3)	0.0545 (4)	0.0666 (4)	0.0023 (2)	0.0016 (3)	0.0058 (3)
N2	0.0410 (9)	0.0526 (10)	0.0555 (11)	0.0008 (8)	0.0005 (8)	−0.0025 (8)
N1	0.0417 (9)	0.0596 (11)	0.0509 (10)	0.0024 (8)	0.0035 (7)	−0.0017 (8)
O1	0.1079 (17)	0.0666 (12)	0.0679 (14)	−0.0285 (12)	−0.0003 (12)	−0.0015 (11)
C9	0.0459 (12)	0.0556 (13)	0.0621 (14)	−0.0015 (10)	0.0020 (10)	−0.0123 (11)
C14	0.0498 (12)	0.0509 (12)	0.0562 (13)	−0.0018 (9)	0.0134 (10)	0.0014 (10)
N3	0.0610 (13)	0.0635 (14)	0.0759 (15)	0.0105 (11)	−0.0048 (11)	−0.0176 (11)
C7	0.0431 (10)	0.0533 (12)	0.0500 (11)	−0.0014 (9)	0.0066 (9)	−0.0015 (9)
C13	0.0444 (11)	0.0564 (13)	0.0560 (13)	−0.0008 (10)	0.0052 (9)	−0.0058 (11)
C10	0.0422 (11)	0.0541 (12)	0.0552 (12)	−0.0006 (9)	0.0073 (9)	−0.0027 (10)
C11	0.0490 (12)	0.0500 (12)	0.0630 (14)	0.0034 (10)	0.0046 (10)	−0.0039 (10)
C8	0.0485 (12)	0.0516 (13)	0.0614 (14)	0.0038 (10)	0.0057 (10)	−0.0061 (10)
C6	0.0457 (11)	0.0598 (13)	0.0490 (12)	0.0023 (10)	0.0067 (9)	−0.0032 (10)
C12	0.0491 (12)	0.0536 (13)	0.0577 (13)	−0.0037 (10)	0.0004 (10)	−0.0081 (10)
C5	0.0558 (14)	0.0617 (15)	0.0714 (16)	0.0053 (12)	0.0030 (11)	0.0020 (12)
C1	0.0488 (12)	0.0681 (16)	0.0627 (15)	−0.0034 (11)	0.0007 (10)	0.0016 (12)
C2	0.0510 (14)	0.092 (2)	0.0656 (16)	−0.0049 (14)	0.0009 (11)	−0.0072 (14)
C4	0.0709 (17)	0.0776 (19)	0.0721 (18)	0.0199 (15)	0.0007 (13)	0.0035 (14)
C3	0.0559 (15)	0.102 (2)	0.0566 (15)	0.0155 (15)	−0.0005 (11)	−0.0039 (14)

### Geometric parameters (Å, °)

**Table:**

S1—C14	1.690 (2)	C13—H13	0.95 (3)
N2—C14	1.341 (3)	C10—C11	1.396 (3)
N2—N1	1.371 (3)	C11—C12	1.378 (3)
N2—H14	0.92 (3)	C11—H11	0.94 (3)
N1—C13	1.278 (3)	C8—H8	0.97 (3)
O1—H17	0.86 (5)	C6—C1	1.393 (4)
O1—H18	0.77 (5)	C6—C5	1.393 (4)
C9—C8	1.387 (3)	C12—H12	0.96 (3)
C9—C10	1.397 (3)	C5—C4	1.396 (4)
C9—H9	0.97 (3)	C5—H5	0.98 (3)
C14—N3	1.332 (3)	C1—C2	1.390 (4)
N3—H15	0.90 (3)	C1—H1	0.98 (3)
N3—H16	0.86 (4)	C2—C3	1.377 (5)
C7—C8	1.390 (3)	C2—H2	0.98 (4)
C7—C12	1.399 (3)	C4—C3	1.374 (5)
C7—C6	1.480 (3)	C4—H4	1.01 (4)
C13—C10	1.454 (3)	C3—H3	0.99 (3)
			
C14—N2—N1	118.4 (2)	C10—C11—H11	118.6 (17)
C14—N2—H14	119.4 (17)	C9—C8—C7	121.0 (2)
N1—N2—H14	122.1 (17)	C9—C8—H8	118.1 (16)
C13—N1—N2	116.9 (2)	C7—C8—H8	120.9 (16)
H17—O1—H18	113 (4)	C1—C6—C5	117.7 (2)
C8—C9—C10	121.1 (2)	C1—C6—C7	121.3 (2)
C8—C9—H9	120.6 (15)	C5—C6—C7	121.0 (2)
C10—C9—H9	118.1 (15)	C11—C12—C7	121.5 (2)
N3—C14—N2	116.4 (2)	C11—C12—H12	120.0 (16)
N3—C14—S1	122.39 (19)	C7—C12—H12	118.4 (16)
N2—C14—S1	121.19 (18)	C6—C5—C4	120.8 (3)
C14—N3—H15	114.4 (19)	C6—C5—H5	118.1 (18)
C14—N3—H16	120 (2)	C4—C5—H5	121.1 (18)
H15—N3—H16	124 (3)	C2—C1—C6	121.2 (3)
C8—C7—C12	117.7 (2)	C2—C1—H1	120.6 (15)
C8—C7—C6	121.3 (2)	C6—C1—H1	118.1 (15)
C12—C7—C6	121.0 (2)	C3—C2—C1	120.1 (3)
N1—C13—C10	120.4 (2)	C3—C2—H2	120 (2)
N1—C13—H13	122.5 (15)	C1—C2—H2	120 (2)
C10—C13—H13	117.1 (15)	C3—C4—C5	120.4 (3)
C11—C10—C9	117.8 (2)	C3—C4—H4	121 (2)
C11—C10—C13	121.9 (2)	C5—C4—H4	119 (2)
C9—C10—C13	120.3 (2)	C4—C3—C2	119.7 (3)
C12—C11—C10	120.8 (2)	C4—C3—H3	119.5 (19)
C12—C11—H11	120.6 (17)	C2—C3—H3	120.8 (19)
			
C14—N2—N1—C13	−173.4 (2)	C12—C7—C6—C1	35.4 (3)
N1—N2—C14—N3	−2.6 (3)	C8—C7—C6—C5	36.1 (3)
N1—N2—C14—S1	176.85 (16)	C12—C7—C6—C5	−144.8 (3)
N2—N1—C13—C10	179.54 (19)	C10—C11—C12—C7	0.9 (4)
C8—C9—C10—C11	1.9 (4)	C8—C7—C12—C11	0.3 (4)
C8—C9—C10—C13	−178.5 (2)	C6—C7—C12—C11	−178.8 (2)
N1—C13—C10—C11	3.1 (4)	C1—C6—C5—C4	1.7 (4)
N1—C13—C10—C9	−176.4 (2)	C7—C6—C5—C4	−178.1 (2)
C9—C10—C11—C12	−2.0 (4)	C5—C6—C1—C2	−1.9 (4)
C13—C10—C11—C12	178.5 (2)	C7—C6—C1—C2	177.9 (2)
C10—C9—C8—C7	−0.7 (4)	C6—C1—C2—C3	0.7 (4)
C12—C7—C8—C9	−0.4 (4)	C6—C5—C4—C3	−0.3 (4)
C6—C7—C8—C9	178.8 (2)	C5—C4—C3—C2	−1.0 (4)
C8—C7—C6—C1	−143.7 (2)	C1—C2—C3—C4	0.8 (4)

### Hydrogen-bond geometry (Å, °)

**Table:**

*D*—H···*A*	*D*—H	H···*A*	*D*···*A*	*D*—H···*A*
N2—H14···O1	0.92 (3)	1.91 (3)	2.819 (3)	172 (3)
N3—H15···N1	0.91 (3)	2.14 (3)	2.585 (3)	110 (2)
N3—H16···S1^i^	0.86 (4)	2.59 (4)	3.422 (3)	163 (3)
O1—H17···S1^ii^	0.86 (5)	2.44 (5)	3.287 (3)	169 (4)
O1—H18···S1^iii^	0.77 (5)	2.60 (5)	3.352 (3)	164 (5)

Symmetry codes:  (i) −*x*+2, *y*−1/2, −*z*+1/2; (ii) *x*, *y*+1, *z*; (iii) −*x*+2, −*y*, −*z*+1.
